# Transforming berberine into its intestine-absorbable form by the gut microbiota

**DOI:** 10.1038/srep12155

**Published:** 2015-07-15

**Authors:** Ru Feng, Jia-Wen Shou, Zhen-Xiong Zhao, Chi-Yu He, Chao Ma, Min Huang, Jie Fu, Xiang-Shan Tan, Xiao-Yang Li, Bao-Ying Wen, Xi Chen, Xin-Yi Yang, Gang Ren, Yuan Lin, Yangchao Chen, Xue-Fu You, Yan Wang, Jian-Dong Jiang

**Affiliations:** 1 State Key Laboratory of Bioactive Substance and Function of Natural Medicines, Institute of Materia Medica, Chinese Academy of Medical Sciences / Peking Union Medical College, Beijing 100050, China; 2 Beijing Analytical Application Center, Shimadzu (China) Co., Ltd., Beijing 100020, China; 3 Institute of Medicinal Biotechnology, Chinese Academy of Medical Sciences / Peking Union Medical College, Beijing 100050, China; 4 School of Biomedical Sciences, Faculty of Medicine, The Chinese University of Hong Kong, Shatin, N.T., Hong Kong, China

## Abstract

The gut microbiota is important in the pathogenesis of energy-metabolism related diseases. We focused on the interaction between intestinal bacteria and orally administered chemical drugs. Oral administration of berberine (BBR) effectively treats patients with metabolic disorders. However, because BBR exhibits poor solubility, its absorption mechanism remains unknown. Here, we show that the gut microbiota converts BBR into its absorbable form of dihydroberberine (dhBBR), which has an intestinal absorption rate 5-fold that of BBR in animals. The reduction of BBR to dhBBR was performed by nitroreductases of the gut microbiota. DhBBR was unstable in solution and reverted to BBR in intestine tissues via oxidization. Heat inactivation of intestinal homogenate did not inhibit dhBBR oxidization, suggesting the process a non-enzymatic reaction. The diminution of intestinal bacteria via orally treating KK-Ay mice with antibiotics decreased the BBR-to-dhBBR conversion and blood BBR; accordingly, the lipid- and glucose-lowering efficacy of BBR was reduced. Conclusively, the gut microbiota reduces BBR into its absorbable form of dhBBR, which then oxidizes back to BBR after absorption in intestine tissues and enters the blood. Thus, interaction(s) between the gut microbiota and orally administrated drugs may modify the structure and function of chemicals and be important in drug investigation.

The mammalian intestine hosts extremely diverse and vast microorganisms, collectively referred to as the gut microbiota, which mostly comprises four bacterial phyla: the Gram-negative Bacteroidetes and Proteobacteria and the Gram-positive Actinobacteria and Firmicutes^1^. In humans, the gut microbiota consists of more than 10^14^ bacteria and archaea, which cover approximately 1,100 prevalent species^1^. The gut microbiota is considered a “hidden organ” of the body and may be associated with the pathogenesis of diseases such as cardiovascular diseases, obesity, and diabetes^2^,^3^,^4^. The microbiota is also an interesting field of research in drug metabolism, particularly with respect to orally administered drugs because the drug metabolism by gut microbiota may generate metabolites different from those generated by the host organs^5^,^6^. It is particularly important if the metabolites generated by the gut microbiota possess novel features and bioactivities. Thus, the drug metabolism by intestinal microorganisms is considered attractive in pharmaceutical research. Here, we present berberine (BBR) as an interesting drug example to show the significance of the gut microbiota in drug investigations.

BBR (Fig. 1a) is a medicinal alkaloid isolated from *Coptis chinensis* and has been used orally for decades in China as an over-the-counter (OTC) drug to treat diarrhea with good safety^7^. We and others have previously identified BBR as a new medicine for hyperlipidemia and type 2 diabetes because it reduces blood lipids and glucose in patients^7^,^8^ through a multiple-target mechanism involving low-density-lipoprotein receptor (LDLR), insulin receptor (InsR), AMP-activated protein kinase (AMPK), and proprotein convertase subtilisin kexin 9 (PCSK9), among others^7^,^8^,^9^,^10^,^11^. The therapeutic effect of BBR on lipid- and glucose-related metabolic disorders was recently confirmed in a large number of patients by independent clinical groups both in and outside China^12^, demonstrating the botanic compound to be a promising drug. By contrast, BBR exhibits poor water solubility and is presumably very difficult for intestinal epithelial cells to absorb^13^,^14^. However, after its oral administration, BBR was detected in nearly all major organs as well as in urine, with the liver exhibiting the highest concentration^15^, and these findings raise the question of how BBR is absorbed in the intestine. Our recent study on the metabolism of BBR in the intestine has identified the gut microbiota as the most likely answer, at least in part, to this question, and we consider the finding of general interest in drug discovery and investigation.

## Results

### DhBBR is a BBR metabolite generated in the intestinal ecosystem

**Figure 1:**
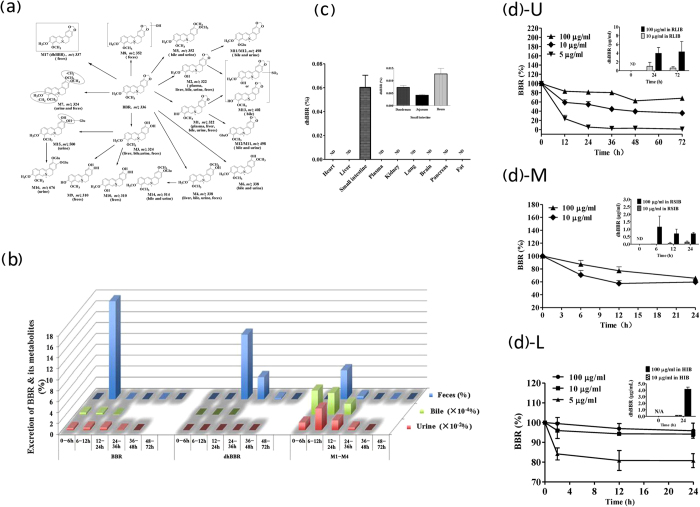
BBR is metabolized into dhBBR in the intestine ecosystem. (**a**) *In vivo* BBR metabolites in SD rats; M17 (*m/z* 337) was identified to be dihydroberberine (dhBBR), a BBR metabolite detected in the feces only; the parentheses indicate the sample in which the compound was detected. (**b**) Excretion of BBR, dhBBR and other BBR metabolites in rat feces, urine and bile after 72 h. (**c**) Distribution of dhBBR in organs of SD rats orally treated with BBR (200 mg/kg); ND: not detectable. (**d**) Conversion of BBR into dhBBR by the gut microbiota *in vitro*; U & insert: in rat large intestinal bacteria (RLIB); M & insert: in rat small intestinal bacteria (RSIB); L & insert: in human intestinal bacteria (HIB) *in vitro*. The Y axis shows the percentage of the administered amount of BBR (n = 6).

To advance our understanding of the *in vivo* fate of BBR after oral administration, we recently investigated the BBR metabolites in the urine, bile and feces in rat. The compound detection was performed using both liquid chromatography coupled with tandem mass spectrometry (LC-MS/MS) and gas chromatography coupled with mass spectrometry (GC-MS). Among the 17 BBR metabolites detected in body excretion, a new metabolite, denoted dihydroberberine (dhBBR), caught our attention (Fig. 1a, in frame). First, dhBBR was detectable in feces but not in the bile and urine samples. Second, among these metabolites, dhBBR was the only one that exhibited a change in the backbone structure (Fig. 1a). We assumed that it might be a metabolite generated by the gut microbiota of rats. We then detected BBR, dhBBR and other BBR metabolites in rat excretions over a 72-h period after the oral administration of BBR. As shown in Fig. 1b, although other major BBR metabolites (M1-M4) were detectable in urine, bile and feces, dhBBR was detected only in feces. The amount of dhBBR reached its peak within 12–24 h and then decreased over time. To further confirm that dhBBR is generated within the intestine ecosystem, rat organs were examined for the presence of dhBBR. As shown in Fig. 1c, 24 h after the oral administration of BBR (200 mg/kg) in rats, dhBBR was detected in intestinal tissue only (including duodenum, jejunum and ileum) but not in other organs. Thus, we considered that rat intestinal bacteria might be the “organ” that converts BBR into dhBBR. We then incubated BBR with a large amount of rat intestinal bacteria *in vitro* and found that the amount of BBR decreased over time (Fig. 1d, U) and that, accordingly, dhBBR became detectable within 24 h of incubation (Fig. 1d, U insert). Next, we investigated samples of the rat small intestine by incubating BBR with bacteria isolated from the small intestine of untreated rats. As shown in Fig. 1d (M), the BBR content decreased over time and dhBBR became detectable within 6 h of incubation, suggesting that the bacteria from the small intestine were also active in the conversion of BBR into dhBBR. We also performed the experiment using human gut microbiota, and the results were similar to those obtained with rat gut bacteria (Fig. 1d, L).

### Intestinal dhBBR was generated by gut microbiota

**Figure 2:**
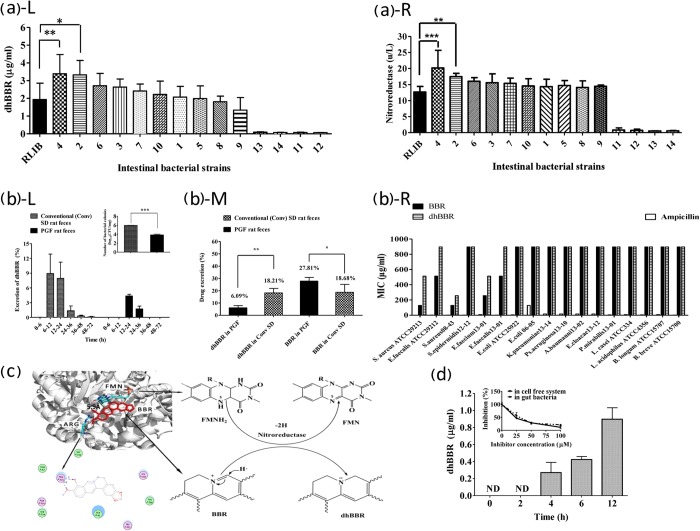
Generation of dhBBR by the gut microbiota. (**a**) BBR was converted *in vitro* into dhBBR by 14 intestinal bacteria strains [*S. aureus* 08-43 (1), *E. faecium* 13-01 (2), *E. faecalis* 13-01 (3), *E. cloacae* 13-12 (4), *E. coli* 06-05 (5), *S. epidermidis* 12-12 (6), *Ps. aeruginosa* 13-10 (7), *K. pneumoniae* 13-14 (8), *P. mirabilis* 13-01 (9), *A. baumannii* 13-02 (10), and *L. casei* (11)*, L. acidophilus* (12), *B. longum* (13), and *B. breve* (14)]. L: dhBBR was produced at different levels by the 14 intestinal bacteria strains (*P < 0.05 and **P < 0.01, n = 6); R: Determination of nitroreductase in the 14 intestinal bacteria strains (***P < 0.001, *P < 0.05, n = 6). (**b**) Oral treatment with antibiotics generated pseudo germ-free (PGF) rats and resulted in a reduced conversion of BBR to dhBBR in the rat intestinal ecosystem; L: dhBBR production was delayed and decreased in the feces of PGF rats compared with conventional SD rats; L insert: oral treatment with antibiotics for 3 days reduced the bacterial colony numbers by 2 logs in the rat feces (***P < 0.001, n = 6); M: the accumulative excretion of BBR and dhBBR in PGF and conventional rats over a period of 72 h (**P < 0.01, *P < 0.05, n = 6); R: MIC of BBR and dhBBR on the 17 intestinal bacteria (24 h); The Y axis shows the percentage of the administered amount of BBR. (**c**) Molecular docking between BBR and nitroreductase, showing the chemical me**c**hanism of BBR reduction by nitroreductase. (**d**) Nitroreductase-mediated BBR reduction resulted in a BBR-to-dhBBR conversion after 4 h; insert: BBR-to-dhBBR conversion was reduced in the presence of the nitroreductase inhibitor 2-IBA (100 μM) in the nitroreductase-containing cell-free incubation mixture or in the rat gut bacteria cultivation mixture (for 12 h); ND: not detectable.

To determine whether the gut microbiota is responsible for the BBR-to-dhBBR conversion, we incubated BBR (50 μg/ml) *in vitro* with 14 intestinal bacterial strains (*Staphylococcus aureus, Enterococcus faecium, Enterococcus faecalis, Enterobacter cloacae, Escherichia coli, Staphylococcus, Pseudomonas aeruginosa, Klebsiella pneumonia, Proteus mirabilis, Acinetobacter baumannii, Lactobacillus casei, Lactobacillus acidophilus, Bifidobacterium longum, and Bifidobacterium breve*) and then measured the amount of dhBBR in the culture. The first 10 strains were clinical isolates and the last four strains are resident intestinal bacteria. As shown in Fig. 2a–L, all 10 of the clinical isolates produced dhBBR after incubation with BBR for 72 h. *Enterobacter cloacae* and *Enterococcus faecium* produced more dhBBR than did the rat feces, which were used as a positive reference, and *Proteus mirabilis* produced less (Fig. 2a). The four resident intestinal bacteria produced dhBBR at a level markedly lower than that obtained with the clinical isolates. The results suggest that the intestinal microbiota appeared to work as a “tissue” to transform BBR into dhBBR. If this is true, oral treatment of rats with antibiotics should reduce dhBBR production by the intestinal bacteria. Thus, SD rats were treated orally with cefadroxil (100 mg/kg), terramycin (300 mg/kg) and erythromycin (300 mg/kg), bid, for 3 days. Two days later, the colon contents were collected for bacterial examination. As shown in Fig. 2b (L insert), oral treatment with the antibiotics for 3 days reduced the bacterial colony in the rat feces by 2 logs, indicating a pseudo germ-free (PGF) intestinal environment in the rats after treatment with antibiotics. BBR (200 mg/kg) was then orally administered to the PGF rats using untreated rats as a control; this step was followed by 72 h of feces collection. BBR and dhBBR in the feces were measured using LC-MS/MS 8040 and GC-MS. Fig. 2b, L shows that dhBBR production was delayed and markedly decreased in the intestine of the PGF rats compared with that in the untreated rats, consistent with the numbers of intestinal bacteria in the PGF rats. In addition, the 72-h-cumulative dhBBR level in the feces of the PGF rats was examined. The accumulated dhBBR was significantly higher in the untreated rats (18.21% of the giving amount) than that in the PGF rats (6.09% of the giving amount, *P* < 0.01); accordingly, the total BBR in the feces of the PGF rats was significantly higher (27.81% of the administered amount) than that in the untreated rats (18.67% of the administered amount, *P* < 0.05, Fig. 2b, M). To evaluate the direct effect of BBR on the gut microbiota, we incubated BBR separately with 17 strains of intestinal bacteria. As shown in Fig. 2b, R, the minimal inhibitory concentration (MIC) values of BBR on the 17 bacteria ranged from 128 to more than 512 μg/ml, which is a concentration close to that of BBR precipitation in the culture medium, indicating that the antibacterial effect of BBR on the gut microbiota was minor. In addition, the antibacterial effect of dhBBR was even weaker than that of BBR in 5 of the 17 intestinal bacteria strains (Fig. 2b, R). Ampicillin was used as the positive control in this experiment.

BBR was converted to dhBBR in a reduction reaction, and nitroreductase and azoreductase are two major reductases that are abundant in the gut microbiota^16^. Because the crystal structures of the reductases have been illustrated and are available in the Protein Data Bank^17^, we performed computer-assistant docking in the initial step of analysis, using the AutoDock Vina v.1.0.2 software. The putative chemical mechanism for BBR reduction by nitroreductase is shown in Fig. 2c. BBR exhibited excellent docking performance at docking onto nitroreductase with a binding free energy of −6.5 kcal/mol. BBR anchored into the binding site of nitroreductase through a strong hydrogen bond interaction with the side chain of Arg-225. The distance between the C atom of the C = N group and the N_5_ atom of FMN was approximately 5.9 Å, which is within the distance good for electron transfer. After binding to nitroreductase in the pocket, BBR obtained H· from the FMN-FMNH_2_ system, and its reduction was activated. The H· from FMNH_2_ moved to the C atom of C = N in BBR, forcing the transfer of one pair of π electrons toward the N atom. The two electrons occupied the orbit of the N atom, forming a lone pair of electrons. The C = N was then transformed into C-N, and BBR was converted into dhBBR (Fig. 2c).

To validate the computational results, the nitroreductase-mediated BBR-to-dhBBR conversion was studied *in vitro*. Following the incubation of BBR with nitroreductase for 0, 2, 4, 6 and 12 h at 37 °C, the amount of dhBBR was examined. As shown in Fig. 2d, dhBBR became detectable after 4 h of incubation of BBR with nitroreductase, and its amount increased over time. Addition of the nitroreductase inhibitor 2-iodosobenzoic acid (2-IBA) into the incubation system significantly inhibited the conversion of BBR to dhBBR, and the inhibition rate reached 88.28% with an increase in the 2-IBA concentration to 100 μM (Fig. 2d, insert), suggesting that the nitroreductase enzymes of the gut bacteria may be an important bacterial enzyme that transforms BBR into dhBBR. Addition of 2-IBA into the incubation system of BBR with the mixture of rat gut bacteria also inhibited dhBBR production by 78.7%, which is in agreement with the results obtained with the cell-free enzymatic reaction. Other bacterial reductases, such as azoreductase (binding free energy of −6.9 kcal/mol), may also be contributors to the conversion. In addition, the correlation between the nitroreductase level in bacteria and BBR-to-dhBBR conversion was examined. In general, as shown in Fig. 2a, L&R, the level of nitroreductase in bacteria was positively correlated with the capability of reducing BBR (into dhBBR), supporting the finding that nitroreductase is at least one of the key enzymes in the conversion of BBR into dhBBR.

### DhBBR exhibited an intestinal absorption rate higher than that of BBR

**Figure 3:**
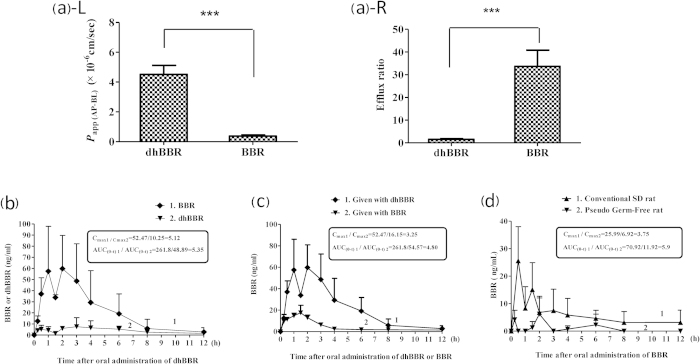
DhBBR has a better intestinal absorption than BBR. (**a**) Absorption of BBR and dhBBR in the Caco-2 cell model; L: The *P*_app (AP-BL)_ of dhBBR in Caco-2 cells was 11.9-fold higher than that of BBR (***P < 0.001); R: The efflux ratio of dhBBR in Caco-2 cells was significantly lower than that of BBR (1.58 vs. 32.39, ***P < 0.001). (**b**) Concentration-time curve for BBR or dhBBR in plasma after dhBBR oral administration (200 mg/kg) to rats (n = 3). (**c**) Concentration-time curve of BBR in plasma after the oral administration of dhBBR (200 mg/kg) or BBR (200 mg/kg) (n = 3). (**d**) Concentration-time curve of BBR in pseudo germ-free rats (generated by the oral administration of antibiotics for 3 days, curve 1) or in conventional SD rats (with no antibiotics, curve 2) after the oral administration of BBR (200 mg/kg, n = 3).

DhBBR in its sulfate form (dihydroberberine sulfate) was previously reported to exhibit better absorption in the intestine as compared with BBR^18^. Here, we focused our investigation on the absorption of the original dhBBR generated by the gut microbiota. First, the absorption of BBR and dhBBR was investigated in the Caco-2 cell model. In this *in vitro* system, dhBBR displayed significantly improved absorption as compared with BBR. The *P*_app (AP-BL)_ (apparent permeability coefficient of AP-BL; AP: apical; BL: basolateral) for dhBBR was 11.9-fold higher than that for BBR (4.51 × 10^−6^ cm/s vs. 0.38 × 10^−6^ cm/s), suggesting that the bioavailability of dhBBR for absorption was markedly increased (Fig. 3a, L). Furthermore, we found that the efflux ratio (ER) for dhBBR was significantly lower than that for BBR (1.58 versus 32.39), indicating a substantial decrease in the cellular efflux of the compound (Fig. 3a, R). Second, the intestinal absorption of dhBBR was studied via *in vivo* experiments in which dhBBR was orally administered to SD rats at a dose of 200 mg/kg. Interestingly, dhBBR was detected at a level less than 10 ng/ml in the plasma (Fig. 3b); instead, a substantial amount of BBR was detected in the blood with an AUC_(0-t)_ value of 261.8 ng/ml*h and a maximum concentration (C_max_) of 52.47 ng/ml (Fig. 3b). The findings suggest that a nearly complete reversion of dhBBR into BBR most likely occurred in the well of the intestine, resulting in a low level of dhBBR and a high level of BBR in blood. The absorption rate of dhBBR (200 mg/kg, oral) was then compared with that of BBR (200 mg/kg, oral). As shown in Fig. 3c, the blood level of BBR in the dhBBR-treated rats exhibited AUC_(0-t)_ and C_max_ values that were 4.8- and 3.25-fold higher than those of the BBR-treated rats, respectively, indicating a higher intestinal absorption of dhBBR as compared with that of BBR.

The results were further tested through another approach. We hypothesized that if dhBBR is the absorbable form of BBR, animals with a low intestinal bacteria content should have less plasma BBR because the level of conversion of BBR to dhBBR in the intestine should be low. We orally treated PGF rats (generated by treatment with antibiotics as mentioned above) with BBR (200 mg/kg) and then measured the blood BBR over a period of 12 h, and antibiotic-free rats administered the same dose of BBR served as control. As shown in Fig. 3d, the blood level of BBR in the PGF rats was much lower than that in those without antibiotics, with a C_max_ ratio of 1/3.75 (PGF rats vs. rats with no antibiotics) and an AUC_(0-t)_ ratio of 1/5.9 (PGF rats vs. rats with no antibiotics), respectively. It seems that the antibiotic-induced decrease in intestinal bacteria resulted in a low level of conversion of BBR into dhBBR in the intestine and thus a low level of BBR in the blood. The results supported the hypothesis presented above. Although dhBBR presented good absorption characteristics, its activity on energy metabolism was lower than that of BBR. As shown in Figure S1 (Supplementary Figure S1), dhBBR remained active for up- regulating LDLR expression but at a level much lower than that of BBR.

### DhBBR reverted to BBR via oxidization in intestine tissues

**Figure 4:**
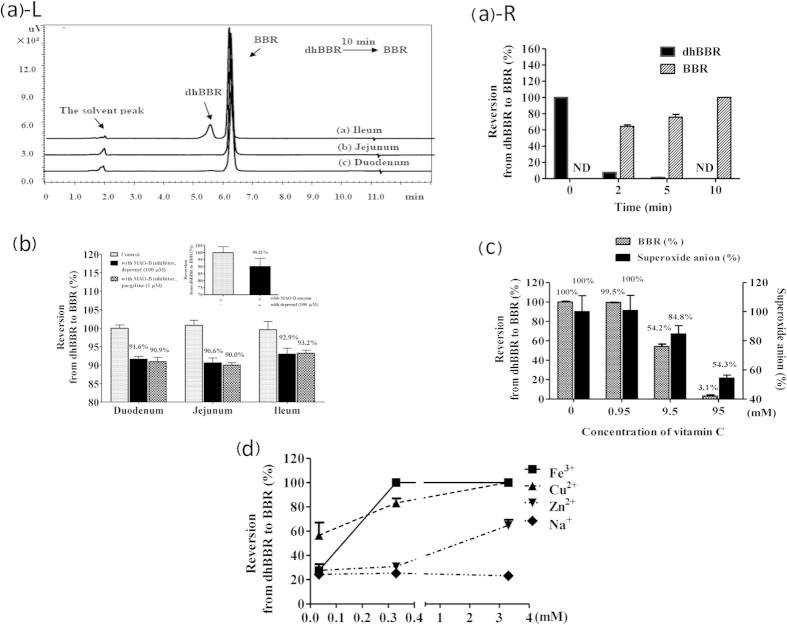
DhBBR reverted to BBR via oxidization in intestine tissue. (**a**) dhBBR-to-BBR reversion in the rat small intestine homogenate (L) and in human intestine microsomes (HIMs, R) (the Y axis shows the percentage of administered amount of dhBBR); ND: not detectable. (**b**) DhBBR-to-BBR reversion by monoamine oxidase-B (MAO-B) in a cell-free system (insert) and homogenates of the rat duodenum, jejunum and ileum; the reversion was slightly inhibited by the MAO-B inhibitor deprenyl (100 μM) or pargyline (1 μM) (the Y axis shows the percentage of administered amount of dhBBR). (**c**) Addition of vitamin C into the homogenate almost terminated the oxidization reaction for reverting dhBBR to BBR, and the content of superoxide anion decreased subsequently (the left Y axis shows the percentage of administered amount of dhBBR; the right Y axis shows the percentage of superoxide anion of control). (**d**) Effect of metal ions (Fe^3+^, Cu^2+^, and Zn^2+^) on catalyzing the oxidization of dhBBR to BBR.

Because dhBBR was barely detectable in the blood, liver and other organs in rats orally treated with BBR (Fig. 1c), we assumed that dhBBR generated by the gut flora in the intestinal cavity must revert to BBR immediately after it enters intestinal wall tissues. Therefore, we incubated dhBBR with homogenates of the small intestine of rats, including homogenates of the duodenum, jejunum or ileum. After a 10-min incubation, dhBBR reversed to BBR almost completely in the duodenum and jejunum homogenate and at a rate of more than 80% in the ileum homogenate (Fig. 4a, L). Human intestinal microsomes, which were rich in CYP450s, also reversed dhBBR to BBR very rapidly (Fig. 4a, R).

Therefore, we investigated the roles of eight subtypes of CYP450 isoforms in the intestine homogenate microsome. The specific CYP450 inhibitors used in the study were furafylline for CYP1A2, ticlopidine for CYP2B6, quercetin for CYP2C8, sulfaphenazole for CYP2C9, nootkatone for CYP2C19, quinidine for CYP2D6, ketoconazole for CYP3A4 and diethyldithiocarbamate (DDC) for CYP2E1. Although all of the recombinant CYP450 isoforms nearly completed the dhBBR-to-BBR reversion (data not shown), the reversion rate in the presence of CYP450 inhibitors was not different from that obtained in the absence of inhibitors in the experiment using pooled human intestinal microsomes (HIMs). This finding suggests that CYP450 might not be critical for reverting dhBBR to BBR. Our next focus was intestinal oxidases, particularly monoamine oxidase (MAO-B)^19^. We incubated dhBBR with MAO-B in a cell-free system first and found that MAO-B may be responsible for approximately 10% reversion from dhBBR to BBR (Fig. 4b, inert). DhBBR was then incubated for 30 min with intestinal homogenate (including duodenum, jejunum or ileum) in the presence or absence of the MAO-B inhibitor deprenyl or pargyline, and this step was followed by BBR detection. As shown in Fig. 4b, incubation of dhBBR with intestinal homogenate resulted in a complete dhBBR-to-BBR reversion, but this reversion was reduced by less than 10% in the presence of a high concentration of the MAO-B inhibitors, which is consistent with the results obtained with the cell-free reaction. It appeared that tissue MAO-B may play a minor role in the dhBBR oxidization course. We then turned our attention to the other factors.

To inactivate proteins within the reaction system, the intestinal homogenate solution was boiled for 2 min. As shown in Figure S2 (Supplementary Figure S2), heat inactivation of the homogenate did not reduce the dhBBR-to-BBR reversion, indicating that the oxidization reaction was mainly a non-enzymatic reaction. Vitamin C, a strong antioxidant, was then added to the reaction solution. As shown in Fig. 4c, the dhBBR-to-BBR reversion was almost completely terminated by vitamin C, suggesting that the oxidization reaction is crucial for the dhBBR-to-BBR reversion in intestinal tissues. Moreover, in this reaction, the content of superoxide anion appeared to be positively correlated with BBR production (Fig. 4c). Metal ions, such as Fe^3+^, Cu^2+^ and Zn^2+^, which are a type of oxidant, appeared to play a positive role in catalyzing the oxidization process, whereas Na^+^ had no effect (Fig. 4d). However, the role of these metal ions (such as Fe^3+^, Cu^2+^ and Zn^2+^) may be weak in the system because the addition of EDTA-2Na reduced the dhBBR-to-BBR oxidization by only 5%.

### Effect of the gut microbiota on the therapeutic efficacy of BBR

**Figure 5:**
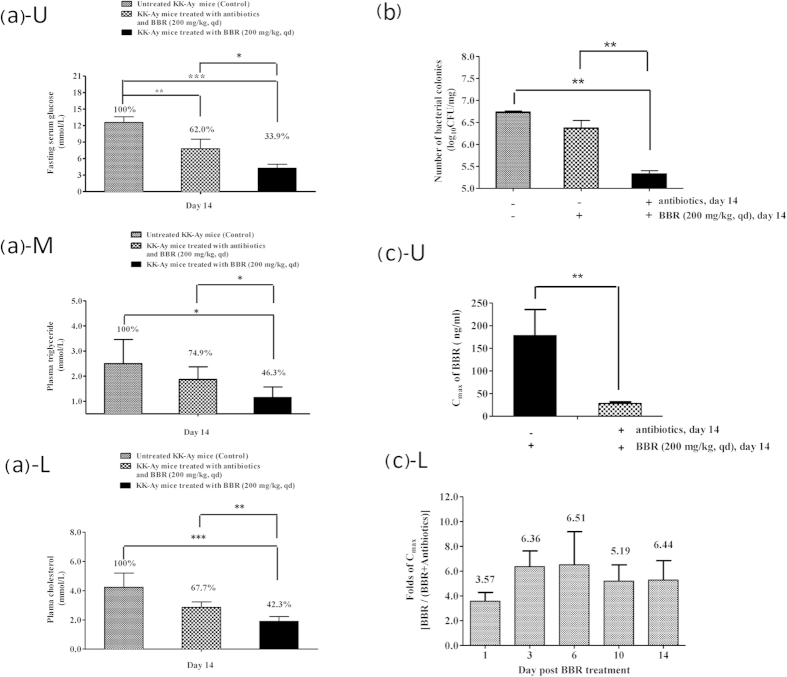
Gut microbiota modulates the therapeutic effect of BBR *in vivo*. (**a**) Fasting blood glucose (Glu), triglyceride (TG) and cholesterol (CHO) in conventional KK-Ay mice or pseudo germ-free KK-Ay mice (n = 6) treated with (or without) BBR for 14 days; U: plasma glucose; M: plasma triglyceride; L: plasma cholesterol. (**b**) Number of intestinal bacteria colonies (in log scale) on day 14 (n = 6). (**c**) Plasma concentration of BBR in KK-Ay mice treated or untreated with antibiotics; U: C_max_ of BBR on day 14; L: folds of C_max_[BBR/(BBR+antibiotics)] on days 1, 3, 6, 10 and 14 post-BBR oral administration. *P < 0.05, **P < 0.01, ***P < 0.001.

To determine whether the gut microbiota alters the therapeutic effects of BBR, we used KK-Ay mice with type 2 diabetes. The mice were either orally pre-treated or untreated with antibiotics for 3 days and then treated with BBR with or without simultaneous treatment with antibiotics. The therapeutic results on day 14 post-BBR treatment are shown in Fig. 5. Compared with the efficacy of BBR in ordinary KK-Ay mice (with no exposure to antibiotics, Group 2), the oral treatment of KK-Ay mice with antibiotics before and during BBR treatment (Group 1) reduced the therapeutic efficacy of BBR on the fasting blood glucose level by 42% [(1−(100–62)/(100–33.9))×100%], the triglyceride level by 53% [(1−(100–74.9)/(100–46.3))×100%] and the cholesterol level by 44% [(1−(100–67.7)/(100–42.3))×100%] (Fig. 5a U, treated vs. untreated, *P* < 0.01, *P* < 0.001; antibiotics plus BBR vs. BBR, *P* < 0.05; Fig. 5a M, treated vs. untreated, *P* < 0.05; antibiotics plus BBR vs. BBR, *P* < 0.05; Fig. 5a L, treated vs. untreated, *P* < 0.001; antibiotics plus BBR vs. BBR, *P* < 0.01). Accordingly, the number of intestinal bacteria colonies in the PGF KK-Ay mice treated with both BBR and antibiotics (Group 1) was significantly lower than that in KK-Ay mice treated with BBR only (Group 2; *P* < 0.01, Fig. 5b). In addition, mice treated with both antibiotics and BBR had fewer intestinal bacteria colonies compared with the untreated controls (Group 1 vs. untreated controls, *P* < 0.01, Fig. 5b).

In addition, we measured the BBR plasma concentrations in KK-Ay mice in the BBR-treated groups with or without antibiotics (Group 1 vs. Group 2). As shown in Fig. 5c (U), on day 14, the C_max_ value of BBR in mice treated with antibiotics (Group 1) was 28.32 ± 3.41 ng/ml whereas in those without antibiotics (Group 2) the BBR C_max_ value was 178.15 ± 57.61 ng/ml, which is 6.4-fold higher than that in Group 1 (*P* < 0.01). In fact, the C_max_ value in Group 2 was 3.5-6.5-fold higher than that in Group 1 throughout the BBR treatment course on days 1, 3, 6, 10 and 14 (Fig. 5c, L). It appeared that the oral administration of antibiotics could lower the level of intestinal bacteria and, accordingly, decrease the absorption of BBR into the blood, reducing its therapeutic efficacy in hyperglycemia and hyperlipidemia.

## Discussion

**Figure 6:**
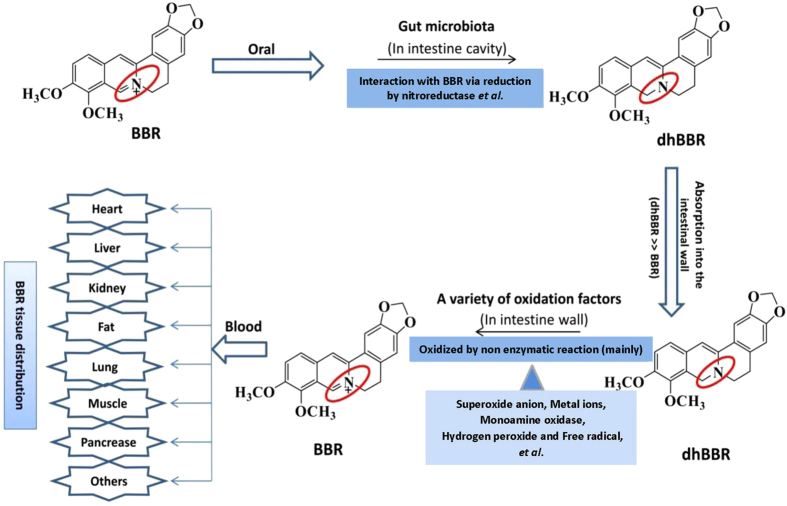
Proposed mechanism of BBR absorption in the intestine.

This study shows that the gut microbiota can act as an “organ” that converts BBR into dhBBR in the intestine via a reduction reaction mediated through bacterial nitroreductase. DhBBR, which was found to be absorbable by the intestinal epithelia, was reverted to BBR (the active form) immediately after entering the intestinal wall tissues. The reversion of dhBBR to BBR via oxidization occurred mainly through a non-enzymatic reaction in intestinal epithelial tissues, with the participation of multi-faceted factors such as superoxide anion and metal ions. These factors in the intestine ecosystem worked in a coordinated manner to complete the structure transformation between BBR (C = N) and dhBBR (C-N). Because the conversion-absorption-reversion process took place entirely in the intestinal environment, BBR, but not dhBBR, is the main chemical form detectable in the blood. Oral administration of antibiotics decreased the amount of intestinal bacteria and thus suppressed BBR absorption, resulting in a low level of BBR in the blood as well as reduced therapeutic efficacy. This study presents an interesting case to show the importance of the gut microbiota in modifying drug bioavailability and therapeutic efficacy. Figure 6 summarizes the role of the gut microbiota in regulating the conversion-absorption -reversion process of BBR in the intestine system.

Nitroreductase in the gut flora plays a very important role in converting BBR into dhBBR. Because this enzyme is abundant in many types of intestinal bacteria^3^, the uptake of BBR in the human intestine should be relatively steady among races and individuals. The physiological condition for dhBBR oxidization is so common that dhBBR molecules readily reverse to BBR, which then enters the bloodstream. In fact, the lipid-lowering and hypoglycemia effects of BBR have been verified in patients by independent clinical groups in China, Europe and America, with similar efficacies (reducing the fasting blood glucose level by 21–36%, the LDL-c level by 10–25% and the triglyceride level by 20–35%)^9^. This finding suggests that the bioavailability of BBR in humans is highly stable. Furthermore, nitroreductase in the gut microbiota may be a rate-limiting step for controlling the amount of BBR entering the blood because the intestinal nitroreductase level is approximately constant at a comparatively flat range in humans. Indeed, BBR exhibits good safety in the clinic and is classified as an OTC drug in China. This study also raises an important issue for patients orally receiving drug treatment together with antibiotics because the uptake rate of drugs (such as BBR) may be modified through antibiotic-mediated changes in the gut microbiota, and, accordingly, the clinical therapeutic efficacy may be altered.

Although dhBBR has a higher intestinal absorption rate than that of BBR, its bioactivity on LDLR and bacteria was lower than that of BBR. The finding agrees with our previous analysis of the structure-activity relationship of BBR, which showed that the 7-position quaternary ammonium and planar structure of the compound are required for LDLR upregulation^20^,^21^,^22^,^23^. It appears that dhBBR is a transient form of BBR in the intestinal lumen, with improved physiochemical characteristics for absorption. Although BBR is considered an antibacterial agent, its activity is directed mainly to dysentery bacillus^24^. The MIC values of BBR for the 17 tested strains were all higher than 128 μg/ml, with most of the values being at least 512 μg/ml, a concentration close to that of BBR precipitation in the testing system. The results showed that the antibacterial effect of BBR was very weak for ordinary intestinal bacteria, and the effect of dhBBR was even weaker. The reduction of BBR to dhBBR by nitroreductase enzymes of the gut microbiota may imply the existence of a self-protection mechanism of intestinal bacteria. In fact, because nitroreductase has broad substrate specificity and reduces groups of compounds, the enzyme is thought to play a role in the general detoxification of bacteria^16^,^25^.

The connection between gut microbes and the pathogenesis of energy metabolic disorders and cardiovascular disease has recently piqued research interest in the physiological function of the gut microbiota in the human body^26^,^27^,^28^,^29^,^30^. This interest has been extended to the interaction between intestinal bacteria and orally administered drugs such as BBR^31^,^32^. Drug metabolism by the intestinal microflora may potentially generate new metabolites with distorted bioactivities or properties; however, the role of the gut microbiota and its influences on orally administered drugs are much less understood. Studies of the functional connections between the gut microbiota and drug pharmacology are even scarcer. The present study on BBR provides a paradigm showing the role of the gut microbiota in modifying drug structure and altering its absorption and therapeutic efficacy. The results may enrich our understanding and knowledge of drug metabolism *in vivo*, drug-drug interaction, mode of action, and druggability prediction. Our findings regarding the bacteria-caused structural modification of BBR in the intestine, as well as the changes in its absorption and therapeutic effects, lead us to suggest that investigation of drug metabolism by the gut microbiota should be a step in drug development.

## Methods

### Chemicals and reagents

BBR was obtained from J&K Scientific Ltd (Beijing, China). Tetrahydropalmatine, as an internal standard, was purchased from the National Institute for Food and Drug Control (Beijing, China). Thalifendine (M1), berberrubine (M2), demethyleneberberine (M3), jatrorrhizine (M4) and dihydroberberine (dhBBR, M17) were obtained from Chengdu Herb Purity Co., Ltd (Chengdu, China). Rutin was purchased from the National Institute for Food and Drug Control (Beijing, China). The purity of the above-mentioned standards was more than 98% (HPLC). HPLC-grade acetonitrile was obtained from J&K Scientific Ltd (Beijing, China). All the other chemicals and reagents were obtained from Sinopharm Chemical Reagent Co., Ltd (Beijing, China) and were of HPLC-grade purity.

Ticlopidine, ketoconazole, β-nicotinamide adenine dinucleotide phosphate (NADP), D-glucose 6-phosphate (G-6-P) and D-glucose-6-phosphate dehydrogenase (G-6-PDH) were purchased from Sigma Chemical Co. (St. Louis, MO, USA). Furafylline, diethyldithiocarbamate (DDC) and sulfaphenazole were obtained from Santa Cruz Biotechnology Co. Ltd. (Shanghai, China). Quercetin, nootkatone and quinidine were purchased from J&K Scientific Ltd. (Beijing, China).

Nitroreductase (≥90%, recombinant, expressed in *Escherichia coli*), monoamine oxidase-B (recombinant, expressed in baculovirus-infected BTI insect cells), and deprenyl (a monoamine oxidase-B inhibitor) were purchased from Sigma-Aldrich Co. (Shanghai, China). 2-Iodosobenzoic (2-IBA, a nitroreductase inhibitor) and pargyline (another monoamine oxidase-B inhibitor) were obtained from J&K Scientific Ltd. (Beijing, China). Human intestinal microsomes (HIMs) were obtained from the Research Institute for Liver Diseases (Shanghai, China).

### Animals

Male Sprague–Dawley (SD) rats (180–200 g) were supplied by the Institute of Laboratory Animal Science, Chinese Academy Medical Sciences (Beijing, China). The animals were housed in cage racks, with a 12-h light/12-h dark cycle (light on from 8:00 AM to 8:00 PM) at ambient temperature (22 °C–24 °C) and 45% relative humidity. The rats were fasted for 12 h before the experiments but during the study had free access to food and water. The research was conducted in accordance with the institutional guidelines and ethics and approved by the Laboratories Institutional Animal Care and Use Committee of the Chinese Academy of Medical Sciences and Peking Union Medical College.

### Instruments

A liquid chromatography instrument coupled to an ion trap time-of-flight mass spectrometer (LC/MS^n^-IT-TOF) from Shimadzu Corporation (Kyoto, Japan) was used to identify the chemical structures of BBR and its possible metabolites. Liquid chromatography with tandem mass spectrometry (LC–MS/MS 8040 or 8050, Shimadzu Corporation, Kyoto, Japan) and gas chromatography with mass spectrometry (GC-MS QP2010, Shimadzu Cooperation, Kyoto, Japan) were used for analysis and quantification of BBR and its metabolites in biological samples.

### Metabolizing BBR by large intestinal bacteria *in vitro*

The method was reported previously^33^. Briefly, colon contents from six rats were pooled, and 5 g of the sample was transferred into a flask containing the anaerobic medium (100 ml). After thorough mixing, the cultures (which contained intestinal bacteria and anaerobic medium) were pre-incubated under anaerobic conditions with a N_2_ atmosphere at 37 °C for 60 min. Rutin (10 μl, 1.0 mg/ml in methanol) was used as a positive reference for intestinal metabolism.

Ten microliters of BBR at different concentrations was added into the fresh human or rat intestinal bacteria cultures (990 μl), with methanol (10 μl) as the negative control. The final concentrations of BBR in the incubation system were 100, 10, and 5 μg/ml. The cultures were incubated at 37 °C for 72 h in the presence or absence of human or rat intestinal bacteria.

After termination of the reaction with acetonitrile (1 ml), 50 μl of the internal standard (tetrahydropalmatine, 2.0 mg/ml in methanol) was added into the BBR samples, which were then mixed for 30 sec and centrifuged at 7,500 g for 15 min. The supernatant was dried under nitrogen flow at room temperature, and the residue was dissolved in 100 μl of methanol and centrifuged at 7,500 g for 15 min. The metabolites were analyzed by LC/MS^n^-IT-TOF using a previously reported method^34^.

GCMS-QP2010 was also used to analyze the BBR metabolites with low polarity. The GC column was an Alltech capillary column (AT^TM^-1701, 30 m × 0.25 mm × 0.25 μm) operated in the splitless mode. The helium carrier flow was 39.7 cm/s under a column head pressure of 68.1 kPa. The oven temperature was initially 50 °C for 2 min, gradually increased to 260 °C at a rate of 8 °C/min, and maintained for 25 min. The injector and detector temperatures were set to 280 °C and 230 °C, respectively. The mass spectra were recorded at a scan range of *m/z* 40 to 800. Structure identification of possible metabolites was based on matching with standard mass spectra available in the Shimadzu GC-MS library.

### Quantification analysis of BBR metabolites modified by the gut microbiota *in vitro* and *in vivo*

A working solution of BBR was prepared at a series of concentrations by diluting the stock solution (10 mg/ml) with methanol to generate the standard curves. LC-MS/MS 8040 was used to quantify BBR and its metabolites transformed by intestinal bacteria^15^.

For the determination of dhBBR obtained after incubation with intestinal bacteria *in vitro*, a working solution of dhBBR was prepared at concentrations of 500, 200, 100, 50, 20, 10 and 1 μg/ml by diluting the stock solution (1 mg/ml) with methanol, which were used to generate the standard curves of dhBBR. Samples from the *in vitro* intestinal bacteria incubation mixture were injected into the GC-MS instrument.

For the analysis of the *in vivo* intestinal metabolites of BBR, six SD rats were orally treated with BBR (200 mg/kg), and their feces were collected 0, 6, 12, 24, 36, 48, and 72 h after treatment. The method used for the preparation of the feces was reported previously^34^. An aliquot of 1 μl was injected into the GC-MS instrument. The level of dhBBR in feces was measured and calculated based on the standard curve obtained with the *in vitro* intestinal bacteria incubation mixture.

To prepare the human samples, we transferred fresh feces (5 g per person) from healthy volunteers (age from 20–25, two males and two females) into a flask containing the anaerobic medium (100 mL)^33^. After thorough mixing, the bacteria were cultured under anaerobic conditions with a N_2_ atmosphere at 37 °C for 60 min. The culture solution of intestinal bacteria was then ready for analysis.

### BBR metabolism in the rat small intestine bacteria

The small intestine contents from ten sacrificed SD rats were obtained and mixed with anaerobic medium to prepare the cultures. BBR was added into the incubation at final concentrations of 100, 50, and 10 μg/ml, and the reaction was continued for 6, 12 or 24 h. Levels of BBR, dhBBR and other BBR metabolites were determinedusing the LC-MS/MS 8040 and GC-MS instruments according to the above-mentioned method.

### Distribution of BBR, dhBBR and other metabolites in the small intestine

Three segments of small intestine (duodenum, jejunum and ileum) were collected 0, 6, 12, 24, 36, and 48 h after the oral administration of BBR in SD rats. The small intestine tissues were washed thoroughly with saline and dried. After being weighed, the samples were homogenized with mixed solution (ethanol: water, 1:1) at a ratio of 1:2 [w(g)/v(ml)]. The samples were centrifuged at 7,500 g for 10 min, and the supernatant was collected and evaporated to dryness in a water bath at 40 °C using a rotary evaporator. The residues of the tissue extraction were dissolved in methanol (250 μl) and vortex-mixed for 5 min. The tissue solution was then centrifuged (at 14,000 g for 5 min), and the supernatant was passed through a 0.22-μm filter. An aliquot of 1 μl of the supernatant was injected into the GC-MS instrument, and a 10-μl aliquot was used for LC-MS/MS analysis. The level of dhBBR was calculated based on the standard curve, and BBR and other metabolites was quantified using the method described above.

### BBR metabolism in the *in vitro* culture of 14 intestinal facultative anaerobes

Ten intestinal facultative anaerobes (clinical isolates)—*Staphylococcus aureus* (*S. aureus*) 08-43, *Enterococcus faecium* (*E. faecium*) 13-01, *Enterococcus faecalis* (*E. faecalis*) 13-01, *Enterobacter cloacae* (*E. cloacae*) 13-12, *Escherichia coli* (*E. coli*) 06-05, *Staphylococcus epidermidis* (*S. epidermidis*) 12-12, *Pseudomonas aeruginosa* (*Ps. aeruginosa*) 13-10, *Klebsiella pneumonia* (*K. pneumoniae*) 13-14, *Proteus mirabilis* (*P. mirabilis*) 13-01 and *Acinetobacter baumannii* (*A. baumannii*) 13-02—were collected from gastrointestinal specimens from patients from hospitals in Beijing between 2006 and 2013. The specimens were identified in the hospitals using the VITEK 2-COMPACT system (bioMerieux, Marcy l’Etoile, France). *Lactobacillus casei (L. casei* ATCC *334), Lactobacillus acidophilus (L. acidophilus* ATCC *4356), Bifidobacterium longum (B. longum* ATCC *15707), and Bifidobacterium breve (B. breve* ATCC *15700)* were purchased from the Microbial Culture Collection Center in Guangdong, China. The bacteria were transferred into a flask containing anaerobic medium. BBR was incubated with the 14 facultative anaerobes at a final concentration of 50 μg/ml at 37 °C for 0, 12, 24, 48 and 72 h. BBR and dhBBR in the culture were analyzed quantitatively using GC-MS and LC-MS/MS 8040.

### Determination of nitroreductase

The detection of nitroreductase was performed using the Human Nitroreductase ELISA kit purchased from Beijing Luyuan Dade Biological Science and Technology Co., Ltd (Beijing, China) according to the manufacturer’s guidelines.

### Pseudo germ-free (PGF) rats and BBR absorption

Male SD rats (180–200 g) were orally treated with cefadroxil (100 mg/kg), terramycin (300 mg/kg) and erythromycin (300 mg/kg) twice a day for 3 days, and pharmacokinetic examination was performed 2 days after final administration. The colon contents of the rats were collected on the first and third days after the final treatment with antibiotics, and the germ-free status was confirmed by culturing fecal samples aerobically on a nutrient agar culture medium. Fecal samples from non-antibiotic-exposed rats served as control samples.

Before oral administration of a single dose of BBR (200 mg/kg), the PGF rats were fasted overnight with free access to water. Blood samples were collected from the posterior orbital venous plexus into a heparinized tube at 0, 0.25, 0.5, 1, 1.5, 2, 3, 4, 6, 8, and 12 h post-BBR treatment and then subjected to the procedure described above. Fecal samples were also collected at 0, 6, 12, 24, 36 and 48 h post-BBR treatment. The samples were stored at −20 °C for further analysis.

### Anti-bacterial test

Seventeen bacterial strains (see above) were used in the susceptibility test for BBR and dhBBR. The bacterial strains, which belong to different species of bacteria, were collected and identified as described above. The minimum inhibitory concentrations (MICs) of BBR and dhBBR after 24 h were determined through the broth microdilution method according to the CLSI guidelines^35^. BBR and dhBBR were tested at a concentration range of 8 to 512 μg/ml. The MIC was defined as the lowest concentration of an agent that prevents turbidity. The experiment was repeated three times. All of the bacterial strains tested in this study (*S. aureus* ATCC 29213, *E. faecalis* ATCC 29212, *E. coli* ATCC 25922, *S. aureus* 08-43, *S. epidermidis* 12-12, *E. faecium* 13-01, *E. faecalis* 13-01, *E. coli* 06-05, *K. pneumoniae* 13-14, *Ps. aeruginosa* 13-10, *A. baumannii* 13-02, *E. cloacae* 13-12, and *P. mirabilis* 13-01) were facultative anaerobes, of which *S. aureus* ATCC 29213, *E. faecalis* ATCC 29212 and *E. coli* ATCC 25922 were standard strains from ATCC (USA) and served as quality controls. *L. casei* ATCC 334*, L. acidophilus* ATCC 4356*, B. longum* ATCC 15707, and *B. breve* ATCC 15700 were also tested in this anti-bacterial experiment. Ampicillin was used as the positive control.

### Molecular docking between BBR and nitroreductase

AutoDock Vina v.1.0.2 software was used to perform the molecular docking of BBR onto nitroreductase, whose crystal structures are available in the Protein Data Bank^17^. The docking parameters were set to the default values. The grid boxes were 20 Å × 20 Å × 20 Å, encompassing the active site cavities. The binding modes of BBR to enzymes were chosen to further optimize the docking conformation according to their binding free energy, distances from conserved water molecules and the flavin mononucleotide (FMN). The simulation results were visualized using the PyMOL Molecular Graphics System Version 1.3 (Schrödinger LLC, New York, NY, USA) and Discovery Studio Visualizer (Accelrys, Inc., San Diego, CA, USA).

### Nitroreductase-mediated BBR reduction

BBR was incubated with nitroreductase in intestinal bacteria cultures. The reaction mixture consisted of BBR (50 μg/ml), an NADPH-regenerating system and nitroreductase (5 μg/ml) in a final volume of 1 ml under the protection of N_2_. After incubation for 0, 2, 4, 6 and 12 h at 37 °C, the reaction was terminated by the addition of 1 ml of ice-cold acetonitrile. The samples were then extracted with 1 ml of ethyl acetate after adding 1 ml of 0.5 M sodium hydroxide solution. The organic phase was evaporated to dryness under a nitrogen flow in a water bath at 40 °C. The residue was dissolved with 200 μl of the mobile phase for further analysis. 2-Iodosobenzoic acid (2-IBA), a specific inhibitor of nitroreductase, was used to verify the role of bacterial nitroreductase.

### Absorption of BBR and dhBBR in Caco-2 cells

A Caco-2 cell assay was conducted using a method reported previously^36^. Stock solutions of dhBBR and BBR were prepared in DMSO at 1 mM, and these were then diluted with HBSS buffer (10 mM HEPES, pH 7.4) to a final concentration of 5 μM for both samples. To determine the rate of drug transport in the apical-to-basolateral direction, 200 μl of the compound working solution was added to the filter well (apical compartment), and 800 μl of HBSS was added to the receiver plate (basolateral compartment). Accordingly, to determine the rate of drug transport in the basolateral-to-apical direction, 800 μl of the compound working solution was added to each well of the receiver plate and 200 μl of HBSS was added to the filter well. The plates were incubated for 30 min at 37 °C with shaking at 150 rpm on a rotary shaker. At the end of the transport period, aliquots of 50 μl were removed directly from the apical and basolateral wells and transferred to wells of new plates. Four volumes of cold methanol containing internal standards were added into each well. The samples were centrifuged at 16,000 g for 15 min. Aliquots of 200 μl of the supernatant were used for the LC-MS/MS analysis.

### Absorption of dhBBR *in vivo*

Five SD rats were fasted for 12 h and then orally administered 200 mg/kg dhBBR. Blood samples (0.5 ml) were obtained from the posterior orbital venous plexus into a heparinized tube at 0, 0.25, 0.5, 1, 1.5, 2, 3, 4, 6, 8, and 12 h, and the samples were then centrifuged at 2,000 g for 5 min. The plasma (100 μl) was precipitated with 100 μl of acetonitrile after addition of 10 μl of the internal standard. BBR served as a control in the study. The plasma concentrations of BBR and dhBBR were determined using LC-MS/MS 8040 and LC-MS/MS 8050.

### Drug effect on hepatic LDLR gene expression

The HepG2 human hepatoma cell line was obtained from the American Tissue Culture Collection (ATCC, USA) and grown in Eagle’s Minimum Essential Medium (GIBCO) supplemented with 10% fetal bovine serum (GIBCO) at 37 °C. For the drug efficacy test, HepG2 cells were cultured for 24 h and then treated with BBR or dhBBR for 12 h. The compounds were freshly prepared in medium prior to use.

The total cellular RNAs were isolated with the SV Total RNA Isolation System (Promega, Madison, WI, USA) following the vendor’s instructions. The total RNAs were reversely transcribed into cDNAs, and quantitative real-time PCR was performed using a 2-Step RT-qPCR System (Promega, Madison, WI, USA), with GAPDH as an internal control. The normalized LDLR mRNA expression levels were plotted as fold levels compared with the untreated control. The following primers were used: LDLR forward, aggacggctacagctaccc; LDLR reverse, ctccaggcagatgttcacg; GAPDH forward, agccacatcgctcagacac; and GAPDH reverse, gcccaatacgaccaaatcc.

### Reversion from dhBBR to BBR

The rat duodenum, jejunum and ileum were collected separately, washed thoroughly with saline and dried. After being weighed, the tissues were homogenized with saline at a ratio of 1: 2 [w (g) /v (ml)]. DhBBR was dissolved in a mixture of methanol and DMSO at a ratio of 1:1 with a final concentration of 5 mg/ml. Ten microliters of dhBBR (5 mg/ml) was mixed with 1 ml of the homogenates. The samples were then centrifuged at 14,000 g for 5 min, and the supernatants were collected and mixed with an equal volume of ice-cold acetonitrile to terminate the reaction. The samples were centrifuged at 14,000 g for 5 min, and the supernatant was used for the HPLC analysis.

### Transformation of dhBBR in pooled human intestinal microsomes (HIMs)

The typical incubation mixture contained 0.2 mg of HIMs, 100 mM phosphate buffer (pH 7.4), 100 μM dhBBR (dissolved in DMSO) and an NADPH-regenerating system [at a final concentration of 3.3 mM glucose-6-phosphate (G-6-P), 1.3 mM NADP^+^, 0.4 unit/ml glucose-6-phosphate dehydrogenase (G-6-PDH) and 3.3 mM MgCl_2_] in a final volume of 200 μl. After pre-incubation of HIMs with dhBBR for 2 min at 37 °C, the reaction was initiated by the addition of the NADPH-regenerating system. The mixture was incubated at 37 °C with exposure to air for 2, 5 and 10 min and was terminated by the addition of 200 μl of ice-cold acetonitrile. Then, 20 μl of the internal standard was added to the mixture, and the mixture was centrifuged at 14,000 g for 5 min. Fifty microliters of the supernatant was injected into the HPLC instrument for analysis. The control samples were incubated under the same conditions but without HIMs.

### Oxidization of dhBBR by monoamine oxidase B (MAO-B)

The reaction system consisted of dhBBR (100 μM), MAO-B (0.2 mg/ml) and phosphate-buffered saline (PBS, pH = 7.4) in a final volume of 200 μl. The MAO-B was pre-incubated via centrifugation at 850 rpm at 37 °C for 5 min, and this step was followed by the addition of dhBBR. The reaction was terminated after 30 min by adding an equal volume of ice-cold acetonitrile and centrifuging at 14,000 g for 5 min. Ten microliters of the supernatant was injected into the HPLC instrument for analysis. Selective MAO-B inhibitors (deprenyl, 100 μM or pargyline, 1 μM)^37^,^38^ were used in the tests.

### Effect of vitamin C on dhBBR-to-BBR transformati*on*

The rats (n = 3) were sacrificed to obtain fresh small intestine homogenate. The homogenate was then boiled for 2 min to inactivate all of the enzymes. After pre-incubation at 37 °C for 2 min, 5 μl of dhBBR (10 mM, dissolved in DMSO) was added into the inactivated homogenate, and fresh homogenate (active one) was used as a reference. The sample was mixed thoroughly and centrifuged at 16,000 g for 5 min. The supernatant was transferred into new tubes, treated with 500 μl of acetonitrile, and then centrifuged. Twenty-microliter aliquots were used for the HPLC analysis.

To examine the oxidative reaction, 100 μl of vitamin C was pre-incubated with the inactivated homogenate; the final concentrations of vitamin C were 95, 9.5 and 0.95 mM. The superoxide anion in the sample was detected with an O_2_^−^ ELISA kit (Beijing Biolab Co., Ltd., Beijing, China; lot: 201404) following the manufacturer’s instructions.

### Gut microbiota modulates the therapeutic effects of BBR on hyperglycemia and hyperlipidemia

KK-Ay mice with type 2 diabetes (female, 12 ± 1 weeks of age) were purchased from the Institute of Laboratory Animal Sciences, Chinese Academy of Medical Sciences (Beijing, China). The mice were maintained at room temperature (23 ± 2 °C) with 50% moisture and exposed to a 12-h light/12-h dark cycle, and food and water were provided ad libitum. Antibiotics at the doses described above were orally administered to KK-Ay mice for 3 days before the BBR treatment course, and the pseudo germ-free (PGF) state of the experimental mice was confirmed (see above).

Four groups (n = 6 for each group) were used in the experiment: Group1, PGF KK-Ay mice orally treated with antibiotics (qd, at the dosage described above) plus BBR (200 mg/kg, qd) for 2 weeks; Group 2, untreated KK-Ay mice orally treated with BBR (200 mg/kg, qd) for 2 weeks; Group 3, PGF KK-Ay mice orally treated with antibiotics (qd, at a dosage identical to that for Group 1) for 2 weeks; Group 4, untreated KK-Ay mice. The antibiotics were administered in the morning and BBR was administered in the afternoon. All the animals were fed a high-fat diet. The germ-free state of Groups 1 and 3 was confirmed on days 7 and 14 of the treatment course using the same bacterial analytical method described above. All of the mice were euthanized on day 14, and blood samples were taken to measure the fasting serum glucose (Glu), triglyceride (TG) and cholesterol (CHO) using Glu, TG and CHO reagent kits (Biosino Bio-Technology and Science INC, Beijing China), respectively. C57BL/6J mice were used as a wild-type reference. The body weights of the mice before, during and after treatments were recorded for 14 days.

Blood samples of the mice in Groups 1 and 2 were collected into a heparinized tube 30 min after the oral administration of BBR. The samples were taken at days 1, 3, 6, 10 and 14 of the BBR treatment course. The samples were centrifuged at 10,000 g for 5 min, and the plasma was collected and stored at −80 °C before analysis.

### Effect of ions on the dhBBR-to-BBR transformatio*n*

dhBBR was added into the reaction solution containing FeCl_3_, CuCl_2_, ZnCl_2_, and NaCl (at concentrations of 0.033, 0.333 and 3.33 mM, respectively). Ethylenediaminetetraacetic acid disodium salt (EDTA-2Na, 10 mM), a chelating agent for metal ions, was used in the test.

### Statistical analysis

The statistical analyses were conducted using two-way ANOVA and Student’s *t*-test with GraphPad Prism Version 5 (GraphPad Software, CA, USA). The data are expressed as the means ± standard deviation, and *p* values less than 0.05 were considered statistically significant.

## Additional Information

**How to cite this article**: Feng, R. *et al.* Transforming berberine into its intestine-absorbable form by the gut microbiota. *Sci. Rep.* **5**, 12155; doi: 10.1038/srep12155 (2015).

## Supplementary Material

Supplementary Information
