# Supplementary material for: Transforming berberine into its intestine-absorbable form by the gut microbiota
Source: Sci Rep. 2015 Jul 15;5:12155. doi: 10.1038/srep12155 (PMC4502414; doi:10.1038/srep12155)
Supplement: Supplementary Information [file srep12155-s1.doc]

**Transforming berberine into its intestine-absorbable form by the gut microbiota**

Ru Feng^1, #^, Jia-Wen Shou^1, #^, Zhen-Xiong Zhao^1, #^, Chi-Yu He^1^, Chao Ma^2^, Min Huang^1^, Jie Fu^1^, Xiang-Shan Tan^1^, Xiao-Yang Li^1^, Bao-Ying Wen^1^, Xi Chen^1^, Xin-Yi Yang^3^, Gang Ren^3^, Yuan Lin^1^, Yangchao Chen^4^, Xue-Fu You^3^, Yan Wang^1, *^, Jian-Dong Jiang^1, 3, *^

^1^ State Key Laboratory of Bioactive Substance and Function of Natural Medicines, Institute of Materia Medica, Chinese Academy of Medical Sciences / Peking Union Medical College, Beijing 100050, China; ^2^ Beijing Analytical Application Center, Shimadzu (China) Co., Ltd., Beijing 100020, China; ^3^ Institute of Medicinal Biotechnology, Chinese Academy of Medical Sciences / Peking Union Medical College, Beijing 100050, China. ^4^ School of Biomedical Sciences, Faculty of Medicine, The Chinese University of Hong Kong, Shatin, N.T., Hong Kong, China.

#These authors made equal contribution to this work.

*Correspondence: Dr. Yan Wang, Tel.: +86 10 63165238, E-mail address: [wangyan@imm.ac.cn](mailto:wangyan@imm.ac.cn); and Dr. Jian-Dong Jiang, Tel.: +86 10 83160005; E-mail address: [jiang.jdong@163.com](mailto:jiang.jdong@163.com).

**Supplementary information**

**Supplementary figure legends and figures**

**Figures legends**

Figure S1: Reduced effect of dhBBR in up-regulating LDLR mRNA in HepG2 cells (treated vs. control, **P*<0.05, ***P*<0.01).

Figure S2: Heat inactivation of the rat homogenate did not reduce dhBBR-to-BBR reversion (the Y axis shows the percentage of the administered amount of dhBBR).

Figure S1

Figure S2
